# Sensitivity to angiotensin II dose in patients with vasodilatory shock: a prespecified analysis of the ATHOS-3 trial

**DOI:** 10.1186/s13613-019-0536-5

**Published:** 2019-06-03

**Authors:** Kealy R. Ham, David W. Boldt, Michael T. McCurdy, Laurence W. Busse, Raphael Favory, Michelle N. Gong, Ashish K. Khanna, Stefan N. Chock, Feng Zeng, Lakhmir S. Chawla, George F. Tidmarsh, Marlies Ostermann

**Affiliations:** 1 Division of Critical Care Medicine, Regions Hospital, University of Minnesota, 640 Jackson Street, St. Paul, MN 55101 USA; 2 Division of Critical Care, Department of Anesthesiology, University of California, Los Angeles, Los Angeles, CA USA; 3 Department of Medicine, University of Maryland School of Medicine, Baltimore, MD USA; 4 Department of Medicine, Emory University, Atlanta, GA USA; 5 Critical Care Center, CHU Lille, Lille, France; 6 Lille Inflammation Research Center, University of Lille School of Medicine, Lille, France; 7 Department of Medicine, Montefiore Healthcare Center, Albert Einstein College of Medicine, Bronx, NY USA; 8 Department of Anesthesiology, Section on Critical Care Medicine, Wake Forest School of Medicine, Winston-Salem, NC & Outcomes Research Consortium, Cleveland, OH USA; 9 Department of Surgery, Sunrise Hospital, Las Vegas, NV USA; 10 La Jolla Pharmaceutical Company, San Diego, CA USA; 11 Department of Critical Care, Guy’s and St. Thomas Hospital, King’s College London, London, UK

**Keywords:** Shock, Hypotension, Angiotensin II, Dose response, Septic shock

## Abstract

**Background:**

Early clinical data showed that some patients with vasodilatory shock are responsive to low doses of angiotensin II. The objective of this analysis was to compare clinical outcomes in patients requiring ≤ 5 ng kg^−1^ min^−1^ angiotensin II at 30 min (≤ 5 ng kg^−1^ min^−1^ subgroup) to maintain mean arterial pressure (MAP) ≥ 75 mmHg versus patients receiving > 5 ng kg^−1^ min^−1^ angiotensin II at 30 min (> 5 ng kg^−1^ min^−1^ subgroup). Data from angiotensin II-treated patients enrolled in the ATHOS-3 trial were used.

**Results:**

The subgroup of patients whose angiotensin II dose was down-titrated from 20 ng kg^−1^ min^−1^ at treatment initiation to ≤ 5 ng kg^−1^ min^−1^ at 30 min (79/163) had significantly lower endogenous serum angiotensin II levels and norepinephrine-equivalent doses and significantly higher MAP versus the > 5 ng kg^−1^ min^−1^ subgroup (84/163). Patients in the ≤ 5 ng kg^−1^ min^−1^ subgroup were more likely to have a MAP response at 3 h versus those in the > 5 ng kg^−1^ min^−1^ subgroup (90% vs. 51%, respectively; odds ratio, 8.46 [95% CI 3.63–19.7], *P* < 0.001). Day 28 survival was also higher in the ≤ 5 ng kg^−1^ min^−1^ subgroup versus the > 5 ng kg^−1^ min^−1^ subgroup (59% vs. 33%, respectively; hazard ratio, 0.48 [95% CI 0.28–0.72], *P* = 0.0007); multivariate analyses supported the survival benefit in patients with lower angiotensin II levels. The ≤ 5 ng kg^−1^ min^−1^ subgroup had a more favorable safety profile and lower treatment discontinuation rate than the > 5 ng kg^−1^ min^−1^ subgroup.

**Conclusions:**

This prespecified analysis showed that down-titration to ≤ 5 ng kg^−1^ min^−1^ angiotensin II at 30 min is an early predictor of favorable clinical outcomes which may be related to relative angiotensin II insufficiency.

**Electronic supplementary material:**

The online version of this article (10.1186/s13613-019-0536-5) contains supplementary material, which is available to authorized users.

## Background

Shock is characterized by inadequate organ perfusion, which, if not rapidly corrected, leads to multiorgan failure and death [1]. International guidelines recommend catecholamines as first-line vasopressor agents [2, 3]. Outcomes remain unacceptable because approximately half of patients with vasodilatory shock do not survive beyond 28 days [4–7]. Additionally, treatment with norepinephrine may cause immunosuppression and seriously complicate the clinical course in patients with septic shock, the most common form of shock [8, 9]. High-dose catecholamines are associated with worse outcomes and adverse events in patients who require higher doses to achieve targeted hemodynamic parameters [9, 10]. An alternative noncatecholamine agent, vasopressin [3], is effective in some patients and may allow the lowering of catecholamine dose, but it has not been shown to improve survival [3]. Further, fewer than 50% of patients in shock generate a blood pressure response to treatment with vasopressin [11], and vasopressin may cause cardiac toxicity, heart failure, and mesenteric ischemia at higher doses [3, 12].

Angiotensin II is a naturally occurring peptide with strong vasopressor activity [13]. A pilot study indicated that the addition of synthetic human angiotensin II to catecholamine and vasopressin therapy maintained mean arterial pressure (MAP) in patients with vasodilatory shock, while allowing for a reduction in catecholamine dosing [14]. In addition, the pilot study noted that 20% of treated patients were exquisitely sensitive to angiotensin II. Given the risk of higher-dose catecholamines, the ability to approach or restore normal hemodynamic function with low doses of systemic vasopressor(s) is an important clinical goal.

Following the pilot study, the randomized, double-blind, phase 3 Angiotensin II for the Treatment of High-Output Shock (ATHOS-3) trial (ClinicalTrials.gov, NCT02338843) demonstrated that angiotensin II significantly increased MAP and provided a catecholamine-sparing effect versus placebo in patients with catecholamine-resistant vasodilatory shock [15]. In this study, patients receiving a stable dose of vasopressor were treated with a starting dose of 20 ng kg^−1^ min^−1^ angiotensin II that could be adjusted every 5 min to achieve a MAP of ≥ 75 mmHg by hour 3. By 30 min, the angiotensin II dose had been down-titrated from 20 to ≤ 5 ng kg^−1^ min^−1^ for 79 of 163 (48%) patients in the treatment arm; analyses comparing this population with those with a dose of > 5 ng kg^−1^ min^−1^ at 30 min were prespecified. In a study by Fliser et al. [16], dosing with 1.5 ng kg^−1^ min^−1^ of angiotensin II resulted in serum levels of ~ 50 pg/mL, just beyond the upper physiologic range for angiotensin II; therefore, administration of 1.5 to 5 ng kg^−1^ min^−1^ angiotensin II is expected to result in serum levels of angiotensin II that approximate physiologic levels in a normotensive individual [16, 17]. Doses of > 5 ng kg^−1^ min^−1^ are expected to result in serum levels in the pharmacologic range [16–18].

We hypothesized that patients most responsive to angiotensin II may have had a defect in angiotensin-converting enzyme function resulting in diminished angiotensin II levels. We further hypothesized that these low angiotensin II levels could be easily corrected to normal physiologic levels by direct exogenous supplementation with the low dose of angiotensin II.

To further characterize the clinical benefit of angiotensin II and to explore these hypotheses, we compared various efficacy and safety parameters in patients receiving angiotensin II in ATHOS-3 who were down-titrated to doses ≤ 5 ng kg^−1^ min^−1^ angiotensin II at 30 min versus patients who required > 5 ng kg^−1^ min^−1^ angiotensin II at 30 min [15].

## Methods

### Study design

Complete details of the ATHOS-3 study (registered January 2015, https://clinicaltrials.gov/ct2/show/NCT02338843; principal investigator: George Tidmarsh) have been previously reported [15]. Briefly, ATHOS-3 was a placebo-controlled study that evaluated the safety and efficacy of angiotensin II as an addition to background vasopressor therapy in patients with catecholamine-resistant vasodilatory shock. Randomization was stratified according to MAP at screening (< 65 mmHg vs. ≥ 65 mmHg) and Acute Physiology and Chronic Health Evaluation II (APACHE II) score (≤ 30, 31 to 40, vs. ≥ 41) on a scale of 0 to 71, with higher scores indicating greater disease severity.

This registrational study was sponsored by La Jolla Pharmaceutical Company, was conducted under a special protocol agreement with the US Food and Drug Administration, and was approved by the appropriate national authorities and local research ethics boards. The study was conducted in accordance with current Good Clinical Practice Guidelines, applicable local regulations, and the ethical principles described in the Declaration of Helsinki.

### Patients

ATHOS-3 enrolled adult patients (aged ≥ 18 years) with vasodilatory shock, defined as central venous oxygen saturation > 70% with central venous pressure > 8 mmHg *or* cardiac index > 2.3 L min^−1^ m^2^, with catecholamine-resistant hypotension (patients receiving > 0.2 µg kg^−1^ min^−1^ of norepinephrine or norepinephrine-equivalent dose for 6 to 48 h prior to enrollment to maintain a MAP of 55 to 70 mmHg) after adequate fluid resuscitation.

### Treatment

Enrolled and consented patients were randomly assigned to angiotensin II or saline placebo. The starting dose of angiotensin II was 20 ng kg^−1^ min^−1^ and could be adjusted every 5 min to achieve a MAP of ≥ 75 mmHg by hour 3. The maximum permitted dose of angiotensin II from hour 0 to 3 was 200 ng kg^−1^ min^−1^. Standard-of-care vasopressors were held constant unless increases were proscribed for safety reasons. From hours 3 to 48, dose of study drug could be adjusted to 1.25 to 40 ng kg^−1^ min^−1^ to maintain a MAP of 65 to 70 mmHg, and standard-of-care vasopressors could be down-titrated. There was a protocol-mandated down-titration of angiotensin II at hour 48. If, upon discontinuation, the subjects’ cardiovascular (CV) Sequential Organ Failure Assessment (SOFA) score was 4, the investigator could restart treatment. During this treatment phase, the dose adjustment criteria remained the same as previously. Angiotensin II was down-titrated gradually, by ≤ 10 ng kg^−1^ min^−1^ every 15 min, when being discontinued and standard-of-care vasopressors were titrated according to institutional protocols. The protocol-specified maximum duration of angiotensin use was 7 days.

### Outcomes

Efficacy outcomes included the proportion of patients who achieved a MAP response at hour 3, change in norepinephrine-equivalent dose from baseline, mortality to day 28, and safety. The MAP response at hour 3 was defined as achieving ≥ 75 mmHg or an increase of ≥ 10 mmHg from baseline at hour 3 without an increase in standard-of-care vasopressors prior to hour 3.

### Statistical methods

The difference in outcomes based on angiotensin II dose at 30 min was a prespecified analysis. No patients discontinued treatment prior to 30 min, and all 163 patients receiving angiotensin II were included.

The association of angiotensin II dose with efficacy parameters was evaluated using univariate and multivariate analyses to adjust for potential imbalances between the two dose groups. Time-to-event endpoints including all-cause survival were summarized using Kaplan–Meier estimates, and unadjusted group comparisons were conducted using the log-rank test. Multivariate time-to-event analyses were conducted using proportional hazards modeling. Binary outcomes (e.g., hour 3 MAP responders) were analyzed by chi-square test for unadjusted analyses and by logistic regression for multivariate analyses.

For multivariate analyses, the following dichotomized baseline covariates were adjusted for age ≥ 65 years, sex, MAP < 65 mmHg, APACHE II score > 30, albumin level < 2.5 g/dL, Model for End-Stage Liver Disease score ≥ 30, chest radiograph finding of acute respiratory distress syndrome (ARDS), and norepinephrine-equivalent dose ≥ 0.5 μg kg^−1^ min^−1^. Additional analyses were conducted, including baseline angiotensin I less than median and angiotensin II less than median.

## Results

### Baseline demographic and disease characteristics

Of the 163 patients who were randomly assigned to receive angiotensin II, 79 patients (48.5%) were receiving ≤ 5 ng kg^−1^ min^−1^ angiotensin II at 30 min, and 84 patients (51.5%) were receiving > 5 ng kg^−1^ min^−1^ angiotensin II at 30 min. Baseline demographic and disease characteristics of these 2 subgroups are shown in Table 1. In general, patients whose angiotensin II dose had been down-titrated at 30 min to ≤ 5 ng kg^−1^ min^−1^ were more likely to have a higher baseline MAP (*P* < 0.0001) and a lower baseline norepinephrine-equivalent dose (*P* = 0.0049). Patients in the ≤ 5 ng kg^−1^ min^−1^ angiotensin II subgroup were also more likely to have lower endogenous levels of angiotensin I (*P* = 0.0058) and angiotensin II (*P* = 0.0009) at baseline (Table 1).

**Table 1:** Baseline disease and demographic characteristics in analysis population

	Angiotensin II > 5 ng kg^−1^ min^−1^ (*n* = 84)	Angiotensin II ≤ 5 ng kg^−1^ min^−1^ (*n* = 79)
Age, *n* (%)		
< 65 years	47 (56.0)	43 (54.4)
≥ 65 years	37 (44.0)	36 (45.6)
Sex, *n* (%)		
Male	47 (56.0)	45 (57.0)
Female	37 (44.0)	34 (43.0)
Race, *n* (%)		
White	69 (82.1)	66 (83.5)
Nonwhite	15 (17.9)	13 (16.5)
Body mass index, *n* (%)	*n* = 83	*n* = 78
< 18.5 kg/m^2^	3 (3.6)	4 (5.1)
≥ 18.5 to < 25 kg/m^2^	22 (26.5)	21 (26.9)
≥ 25 to < 30 kg/m^2^	20 (24.1)	22 (28.2)
≥ 30 kg/m^2^	38 (45.8)	31 (39.7)
Geographic region, *n* (%)		
Australia/New Zealand	17 (20.2)	11 (13.9)
Europe	10 (11.9)	9 (11.4)
USA/Canada	57 (67.9)	59 (74.7)
Albumin, *n* (%)	*n* = 78	*n* = 76
< 2.5 g/dL	56 (71.8)	47 (61.8)
≥ 2.5 g/dL	22 (28.2)	29 (38.2)
Cause of shock, *n* (%)		
Sepsis	68 (81.0)	59 (74.7)
Likely sepsis	8 (9.5)	12 (15.2)
Vasoplegia	3 (3.6)	7 (8.9)
Other	5 (6.0)	1 (1.3)
Baseline MAP,* *n* (%)		
< 65 mmHg	37 (44.0)	15 (19.0)
≥ 65 mmHg	47 (56.0)	64 (81.0)
Baseline APACHE II score, n (%)		
≤ 30	54 (64.3)	51 (64.6)
31–40	29 (34.5)	21 (26.6)
≥ 41	1 (1.2)	7 (8.9)
Norepinephrine-equivalent dose 6 h before randomization, mean, µg/kg/min (SD)	0.52 (0.301)	0.45 (0.377)
Baseline norepinephrine-equivalent dose,* *n* (%)		
< 0.5 µg/kg/min	50 (59.5)	67 (84.8)
≥ 0.5 µg/kg/min	34 (40.5)	12 (15.2)
Baseline angiotensin I,* *n* (%)	*n* = 74	*n* = 71
Mean (SD)	678.9 (810.8)	618.0 (1332.5)
Median (range)	346.0 (10.5–3730.0)	164 (10.5–9180.0)
< 72.3 pg/mL	12 (16.2)	24 (33.8)
72.3 to < 253 pg/mL	14 (18.9)	19 (26.8)
253 to < 676 pg/mL	25 (33.8)	14 (19.7)
≥ 676 pg/mL	23 (31.1)	14 (19.7)
Baseline angiotensin II,* *n* (%)	*n* = 74	*n* = 70
Mean (SD)	420.8 (680.3)	128.3 (199.1)
Median (range)	157.5 (10.5–3340.0)	61.3 (10.5–1090.0)
< 23.85 pg/mL	14 (18.9)	21 (30.0)
23.85 to < 83.75 pg/mL	11 (14.9)	21 (30.0)
83.75 to < 299.5 pg/mL	25 (33.8)	21 (30.0)
≥ 299.5 pg/mL	24 (32.4)	7 (10.0)
Exposure to angiotensin-converting enzyme inhibitors	7 (8.3)	8 (10.1)
Exposure to angiotensin II receptor blockers	9 (10.7)	2 (2.5)
Radiographic finding of ARDS	23 (27.4)	17 (21.5)
Cardiac index (L/min/m^2^)	*n* = 36	*n* = 33
Mean (SD)	3.5 (1.07)	3.1 (0.73)
Median (range)	3.1 (2.3–6.4)	3.0 (2.1–5.4)
ScvO_2_ (%)	*n* = 59	*n* = 61
Mean (SD)	78.2 (8.14)	77 (9.64)
Median (range)	79.0 (53–99)	75.9 (45–99)
Central venous pressure (mmHg)	*n* = 64	*n* = 62
Mean (SD)	14.3 (5.8)	13.2 (4.1)
Median (range)	13.0 (5–35)	12.0 (6–29)
SOFA score		
Mean (SD)	12.0 (2.8)	11.5 (2.8)
Median (range)	12.0 (5–18)	12.0 (5–18)

ARDS, acute respiratory distress syndrome; MAP, mean arterial pressure; ScvO_2_, central venous oxygen saturation; SD, standard deviation; SOFA, Sequential Organ Failure Assessment

All *n*’s are as in the table header, unless otherwise specified

Exposure to angiotensin-converting enzyme inhibitors and angiotensin II receptor blockers within week prior to starting study

*Statistically significant, *P* < 0.01

### Efficacy in angiotensin II dose groups

#### MAP response at hour 3

**Fig. 1:**
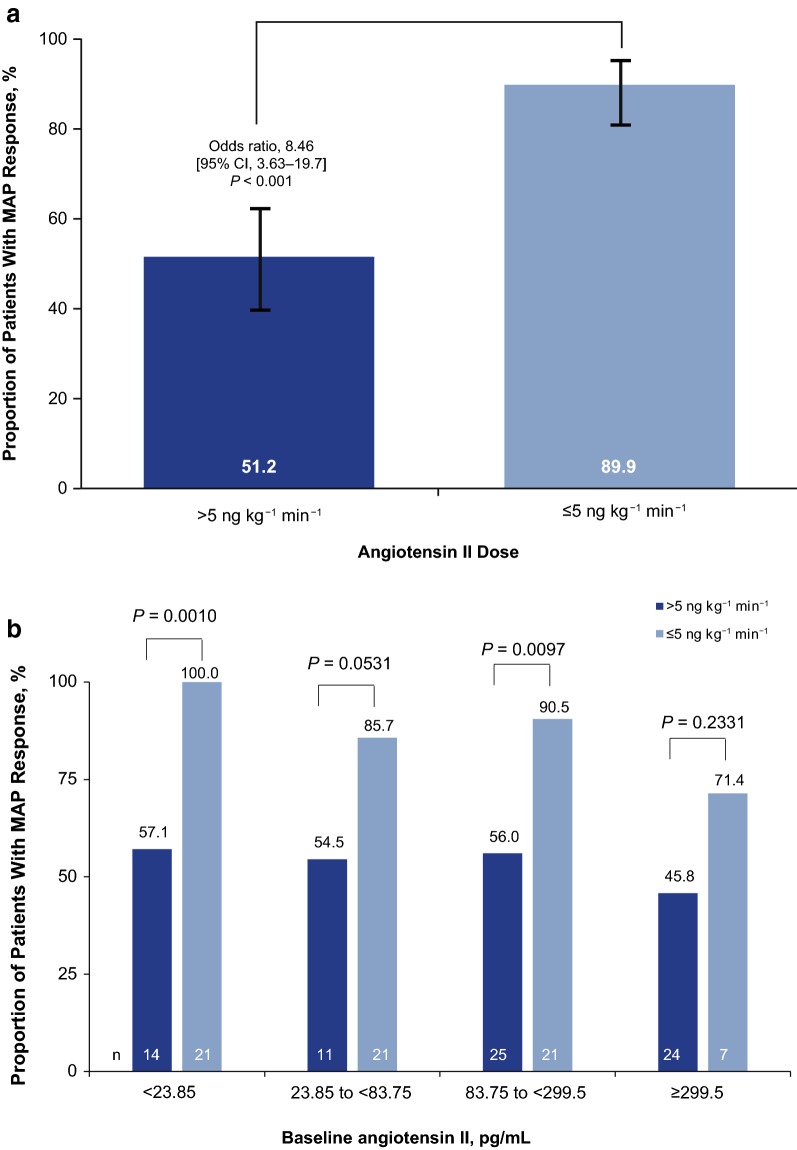
Proportion of patients who had a MAP response at hour 3 by their angiotensin II dose, at 30 min following study initiation; > 5 ng kg^−1^ min^−1^ versus ≤ 5 ng kg^−1^ min^−1^ (**a**). Proportion of patients who had a MAP response at hour 3 by their angiotensin II dose at 30 min and quartiles of baseline angiotensin II (**b**). CI, confidence interval; MAP, mean arterial pressure

A significantly higher proportion of patients receiving ≤ 5 ng kg^−1^ min^−1^ angiotensin II at 30 min achieved a MAP response at hour 3 (≥ 75 mmHg or ≥ 10 mmHg increase in MAP) compared with patients requiring > 5 ng kg^−1^ min^−1^ angiotensin II at 30 min (89.9% [95% CI 81.0–95.5%] vs. 51.2% [95% CI 40.0–62.3%], respectively; unadjusted odds ratio, 8.46 [95% CI 3.63–19.74]; *P* < 0.001; Fig. 1a). Univariate analyses within subgroups of patients showed a consistent trend for higher MAP response in patients receiving 5 ng kg^−1^ min^−1^ angiotensin II at 30 min. This included subgroups defined by the quartiles of baseline angiotensin II (measured in pg/mL). Statistically significant treatment benefits were largest in patients with lower baseline angiotensin II levels (Fig. 1b). Multivariate analyses of MAP response at hour 3, adjusting for baseline disease covariates, were also significant for angiotensin II dose at 30 min (odds ratio, 9.09; 95% CI 3.53–23.4; *P* ≤ 0.0001).

#### Norepinephrine-equivalent doses and SOFA score

Patients in the ≤ 5 ng kg^−1^ min^−1^ angiotensin II subgroup received lower norepinephrine-equivalent doses from hours 3 to 48 (mean [SD] dose 0.16 ng kg^−1^ min^−1^ [0.242]) than patients in the > 5 ng kg^−1^ min^−1^ angiotensin II subgroup (0.51 ng kg^−1^ min^−1^ [0.750]). At hour 48, 41 of 79 (52%) patients in the ≤ 5 ng kg^−1^ min^−1^ angiotensin II subgroup had discontinued all other vasopressors versus 25 of 84 (30%) patients in the > 5 ng kg^−1^ min^−1^ subgroup. Early sensitivity to angiotensin II was also associated with a significantly greater improvement in total SOFA score and CV SOFA score from screening to hour 48 (Table 2).

**Table 2:** Change from screening to hour 48 in total SOFA score and CV SOFA score

	Angiotensin II > 5 ng kg^−1^ min^−1^ (*n* = 84)	Angiotensin II ≤ 5 ng kg^−1^ min^−1^ (*n* = 79)	*P* value
Total SOFA score			
Mean (SD)	2.6 (5.5)	− 0.6 (5.0)	0.002^a^
Median (range)	1.0 (− 6 to 15)	− 2.0 (− 10 to 15)	
CV SOFA score^b^			
Mean (SD)	− 1.33 (1.7)	− 2.2 (1.7)	0.004
Median (range)	0.0 (− 4 to 0)	− 3.0 (–4 to 0)	

CV, cardiovascular; MAP, mean arterial pressure; SOFA, Sequential Organ Failure Assessment; SD, standard deviation

^a^van Elteren Wilcoxon rank test of angiotensin II dose at 30 min stratified by randomization strata for MAP and APACHE II score

^b^All patients had a CV SOFA score of 4 (highest risk) at screening based on inclusion/exclusion criteria. For patients missing a 48-hour assessment, the last observation was carried forward. In the event of death prior to the 48-hour assessment, the patient was assigned a CV SOFA score of 4. The van Elteren Wilcoxon rank test compared angiotensin II with placebo adjusting for randomization strata for MAP and APACHE II score

#### Survival at day 28

**Fig. 2:**
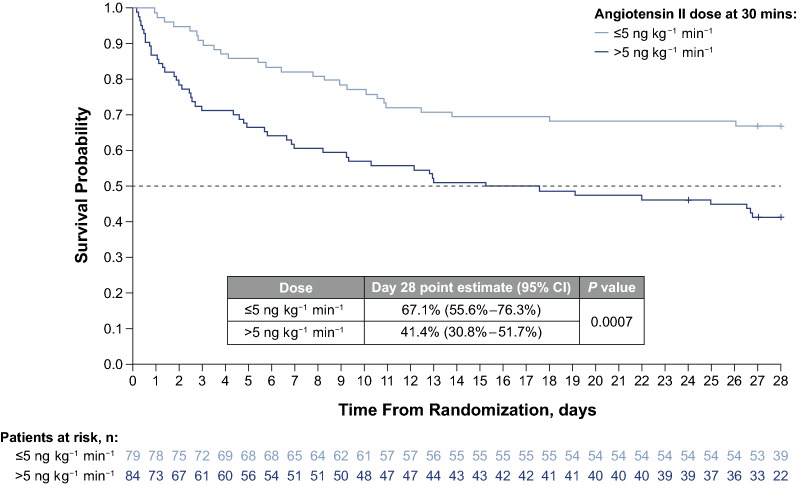
Proportion of patients surviving at day 28 by their angiotensin II dose, at 30 min following study initiation; > 5 ng kg^−1^ min^−1^ versus ≤ 5 ng kg^−1^ min^−1^. CI, confidence interval

Survival at day 28 was 67% (95% CI 56–76%) in patients down-titrated to ≤ 5 ng kg^−1^ min^−1^ angiotensin II at 30 min compared with 41% (95% CI 31–52%) in patients who received > 5 ng kg^−1^ min^−1^ angiotensin II at 30 min (relative risk, 0.45; 95% CI 0.28–0.72; *P* = 0.0007; Fig. 2). Univariate analyses within subgroups of patients showed a consistent trend for higher day 28 survival in patients receiving ≤ 5 ng kg^−1^ min^−1^ angiotensin II at 30 min. For example, survival was significantly higher for patients receiving ≤ 5 ng kg^−1^ min^−1^ angiotensin II regardless of baseline MAP (MAP ≤ 65 mmHg vs. MAP > 65 mmHg) (Table 3). In patient subsets defined by quartiles of baseline angiotensin I or II levels, a higher proportion of patients treated with ≤ 5 ng kg^−1^ min^−1^ angiotensin II were alive at day 28 than patients receiving > 5 ng kg^−1^ min^−1^ angiotensin II. In multivariate analyses to account for imbalances between dose groups, patients treated with ≤ 5 ng kg^−1^ min^−1^ angiotensin II were significantly more likely to survive than those receiving > 5 ng kg^−1^ min^−1^ (relative risk, 0.53; 95% CI 0.32–0.86; *P* = 0.011).

**Table 3:** Univariate analysis of survival at day 28 for baseline covariates significantly different between the angiotensin II dose groups

	Patients, *n*, % (95% CI)		Hazard ratio* (95% CI)	*P* value
	Angiotensin II > 5 ng kg^−1^ min^−1^	Angiotensin II ≤ 5 ng kg^−1^ min^−1^		
Baseline MAP				
≥ 65 mmHg	47, 44.7 (30.2–58.1)	64, 67.2 (54.2–77.2)	0.52 (0.29–0.92)	0.023
< 65 mmHg	37, 37.4 (22.1–52.7)	15, 66.7 (37.5–84.6)	0.38 (0.14–1.00)	0.041
Baseline norepinephrine-equivalent dose				
< 0.5 µg kg^−1^ min^−1^	50, 47.4 (33.0–60.5)	67, 68.7 (56.1–78.3)	0.53 (0.30–0.95)	0.030
≥ 0.5 µg kg^−1^ min^−1^	34, 32.4 (17.6–48.0)	12, 58.3 (27.0–80.1)	0.45 (0.17–1.19)	0.100
Baseline angiotensin I				
< 72.3 pg/mL	12, 41.7 (15.2–66.5)	24, 66.7 (44.3–81.7)	0.49 (0.18–1.35)	0.160
72.3 to < 253 pg/mL	14, 42.9 (17.7–66.0)	19, 57.9 (33.2–76.3)	0.56 (0.21–1.48)	0.234
253 to < 676 pg/mL	25, 39.3 (20.5–57.6)	14, 85.7 (53.9–96.2)	0.18 (0.04–0.80)	0.011
≥ 676 pg/mL	23, 52.2 (30.5–70.0)	14, 50.0 (22.9–72.2)	1.02 (0.40–2.64)	0.962
Baseline angiotensin II				
< 23.85 pg/mL	14, 28.6 (8.8–52.4)	21, 71.4 (47.2–86.0)	0.29 (0.10–0.80)	0.011
23.85 to < 83.75 pg/mL	11, 36.4 (11.2–62.7)	21, 71.4 (47.2–86.0)	0.37 (0.12–1.09)	0.060
83.75 to < 299.5 pg/mL	25, 43.2 (23.5–61.5)	21, 57.1 (33.8–74.9)	0.64 (0.27–1.47)	0.285
≥ 299.5 pg/mL	24, 58.3 (36.4–75.0)	7, 57.1 (17.2–83.7)	1.01 (0.28–3.66)	0.993

CI, confidence interval; MAP, mean arterial pressure

*Hazard ratio/log-rank test result of treatment effect within subgroup

### Treatment exposure and safety in angiotensin II dose groups

In otherwise healthy patients, the plasma concentration of angiotensin II is ~ 5 to 35 pg/mL [19]. In the critically ill patients with distributive shock studied herein, the mean serum concentration of angiotensin II in the > 5 ng kg^−1^ min^−1^ angiotensin II subgroup was 420.8 (± 680.4) pg/mL at baseline and in the ≤ 5 ng kg^−1^ min^−1^ angiotensin II subgroup was 128.3 (± 199.1) pg/mL at baseline. Survival was significantly higher for patients in the ≤ 5 ng kg^−1^ min^−1^ angiotensin II subgroup with relatively low baseline angiotensin II levels (< 23.85 pg/mL) (Table 3).

Patients receiving > 5 ng kg^−1^ min^−1^ angiotensin II at 30 min were more likely to have serious adverse events, moderate to severe adverse events, or adverse events resulting in discontinuation, compared with patients receiving ≤ 5 ng kg^−1^ min^−1^ angiotensin II at 30 min (Table 4). The most commonly reported treatment-emergent adverse events (reported in > 10% of patients in either dosing subgroup) included septic shock, atrial fibrillation, multiorgan failure, hypotension, thrombocytopenia, and hypokalemia (Additional file 1: Table S1).

**Table 4:** Summary of treatment-emergent adverse events (regardless of causality) in either dosing subgroup

	Angiotensin II > 5 ng kg^−1^ min^−1^ (*n* = 84)	Angiotensin II ≤ 5 ng kg^−1^ min^−1^ (*n* = 79)
Any grade, *n* (%)	76 (90.5)	66 (83.5)
Grade 3/4, *n* (%)	64 (76.2)	43 (54.4)
Serious adverse events, *n* (%)	64 (76.2)	35 (44.3)
Adverse events resulting in discontinuation, *n* (%)	19 (22.6)	4 (5.1)
Fatal adverse events, *n* (%)	0	1 (1.3)

## Discussion

Seventy-nine of 163 (48%) patients were down-titrated from an initial angiotensin II dose of 20 ng kg^−1^ min^−1^ to a dose of ≤ 5 ng kg^−1^ min^−1^ at 30 min following initiation of study drug. This subset of patients was more likely to have a MAP response, increased 28-day survival, and better safety profile when considering grade 3/4 and serious AEs. These data may provide health care practitioners an early indication of which patients are most likely to respond to angiotensin II.

Patients in the ≤ 5 ng kg^−1^ min^−1^ angiotensin II subgroup had lower levels of endogenous baseline angiotensin II than their counterparts in the > 5 ng kg^−1^ min^−1^ angiotensin II subgroup. However, the angiotensin II levels for patients with shock are higher than those for normal patients as previous studies have shown levels of angiotensin II in normal adult plasma to range from 5 to 35 pg/mL [19]. The observations seen herein support our hypothesis that patients sensitive to low levels of angiotensin II (≤ 5 ng kg^−1^ min^−1^) are more likely to have an angiotensin II insufficiency. Nonetheless, supplementation with exogenous angiotensin II in “angiotensin II insufficient” patients helped rapidly restore MAP and was associated with improved outcomes. The possible reasons underlying sensitivity to exogenous angiotensin II remain unclear, as patients in the ≤ 5 ng kg^−1^ min^−1^ subgroup had lower baseline angiotensin II levels but also had higher baseline MAP and lower norepinephrine-equivalent doses at baseline. In addition, no difference existed between the > 5 ng kg^−1^ min^−1^ angiotensin II subgroup and the ≤ 5 ng kg^−1^ min^−1^ angiotensin II subgroup in characteristics potentially associated with impaired production/function of angiotensin II (such as prior use of angiotensin-converting enzyme inhibitors, use of angiotensin II receptor blockers, or radiographic evidence of ARDS; Table 1). It is conceivable that angiotensin II sensitivity is linked to endothelial injury, which was not rigorously assessed in the current analyses or the ATHOS-3 study. Across the entire cohort, the delivered dose of exogenous angiotensin II would overcome any inherent angiotensin-converting enzyme defects, so it is likely that attenuation of the MAP response to angiotensin II in the > 5 ng kg^−1^ min^−1^ subgroup may be influenced by other factors, including down-regulation of angiotensin I/II receptors in conditions such as sepsis and/or biofeedback to the production of vasodilatory peptides [20–22]. Although our analysis does not account for all possible mechanisms that could account for the differences in MAP response between the two subgroups, patients receiving ≤ 5 ng kg^−1^ min^−1^ angiotensin II at 30 min represent a subset of patients who may be more likely to safely recover from shock and who can be identified soon after treatment initiation (at 30 min). This observation may allow clinicians to rapidly identify those patients who may benefit most from the continued use of angiotensin II.

Those patients who required ≤ 5 ng kg^−1^ min^−1^ at 30 min had less severe shock (higher baseline MAP) and lower baseline norepinephrine-equivalent doses than those who required > 5 ng kg^−1^ min^−1^. Accordingly, the beneficial effect seen in these patients may support the concept of using angiotensin II earlier in the course of disease. Additionally, in prespecified analyses, these patients exhibited improved survival even after adjusting for baseline differences in shock. Again, this would suggest that the use of angiotensin II at an early stage may be reasonable before a patient progresses to more severe refractory shock at very high norepinephrine-equivalent doses.

The study has several limitations. First, even though this was a prespecified analysis, the subgroups were defined by an on-treatment variable that was likely to be related to drug efficacy and was associated with better prognostic characteristics, not all of which are measured and controllable. However, no patients were discontinued from treatment within 30 min, nor was there selection bias, as all patients receiving angiotensin II were included in the analyses. The on-treatment variable of angiotensin II dose was adjusted based on MAP responsiveness, so higher hour 3 response rates in patients who ultimately required lower hour 3 doses of angiotensin II were expected. However, dose adjustment was made based on prespecified, objective, and easily measurable criteria (i.e., MAP), adding validity to the study findings. The observed improvement in day 28 survival in patients receiving ≤ 5 ng kg^−1^ min^−1^ of angiotensin II is not directly linked to angiotensin II dose, but it is expected that patients with better prognostic characteristics may be more likely to demonstrate immediate responsiveness. As a result, there were significant imbalances in key disease and previous treatment characteristics between the two groups that confound the interpretation of these analyses. For example, patients receiving ≤ 5 ng kg^−1^ min^−1^ angiotensin II had significantly lower baseline norepinephrine-equivalent dose and significantly higher baseline MAP compared with patients in the subgroup receiving > 5 ng kg^−1^ min^−1^. To account for the baseline differences, we performed regression analyses adjusting for the baseline characteristics. However, there may be unmeasured characteristics that were unaccounted for.

## Conclusions

Despite these limitations, this prespecified analysis of ATHOS-3 showed that, for a significant proportion of patients with vasodilatory shock, a low dose of angiotensin II may be effective and is safe, and may provide an early indication of which patients are more likely to respond to angiotensin II. Larger, prospective studies are needed to understand the reasons underlying the phenomenon of hyper-responsiveness to low doses of angiotensin II and to identify all responders. Such information would be informative for clinical decision-making as well as help improve emergency management of patients with vasodilatory shock.

## Additional file

**Additional file 1: Table S1.** Summary of Treatment-Emergent Adverse Events (regardless of causality) Reported in >5% of Patients in Either Dosing Subgroup.

## Data Availability

The datasets used and analyzed during the current analyses are available from the corresponding author on reasonable request.

## Abbreviations

APACHE II: Acute Physiology and Chronic Health Evaluation II
ARDS: acute respiratory distress syndrome
ATHOS-3: Angiotensin II for the Treatment of High-Output Shock
CI: confidence interval
MAP: mean arterial pressure
SD: standard deviation
SOFA: Sequential Organ Failure Assessment

## References

[1] Vincent JL, De Backer D (2013). Circulatory shock. N Engl J Med.

[2] Avni T, Lador A, Lev S, Leibovici L, Paul M, Grossman A (2015). Vasopressors for the treatment of septic shock: systematic review and meta-analysis. PLoS ONE.

[3] Dellinger RP, Levy MM, Rhodes A, Annane D, Gerlach H, Opal SM, Sevransky JE, Sprung CL, Douglas IS, Jaeschke R, Osborn TM, Nunnally ME, Townsend SR, Reinhart K, Kleinpell RM, Angus DC, Deutschman CS, Machado FR, Rubenfeld GD, Webb S, Beale RJ, Vincent JL, Moreno R (2013). Surviving Sepsis Campaign: international guidelines for management of severe sepsis and septic shock, 2012. Intensive Care Med.

[4] De Backer D, Biston P, Devriendt J, Madl C, Chochrad D, Aldecoa C, Brasseur A, Defrance P, Gottignies P, Vincent JL, Investigators SI (2010). Comparison of dopamine and norepinephrine in the treatment of shock. N Engl J Med.

[5] Andreis DT, Singer M (2016). Catecholamines for inflammatory shock: a Jekyll-and-Hyde conundrum. Intensive Care Med.

[6] Hessler M, Arnemann PH, Seidel L, Kampmeier T, Rehberg S, Ertmer C (2017). ATHOS-3 and the knights of the round table–the search for the holy grail of vasopressors. J Thorac Dis.

[7] Schmittinger CA, Torgersen C, Luckner G, Schroder DC, Lorenz I, Dunser MW (2012). Adverse cardiac events during catecholamine vasopressor therapy: a prospective observational study. Intensive Care Med.

[8] Stolk RF, van der Poll T, Angus DC, van der Hoeven JG, Pickkers P, Kox M (2016). Potentially inadvertent immunomodulation: norepinephrine use in sepsis. Am J Respir Crit Care Med.

[9] Yamamura H, Kawazoe Y, Miyamoto K, Yamamoto T, Ohta Y, Morimoto T (2018). Effect of norepinephrine dosage on mortality in patients with septic shock. J Intensive Care.

[10] Martin C, Medam S, Antonini F, Alingrin J, Haddam M, Hammad E, Meyssignac B, Vigne C, Zieleskiewicz L, Leone M (2015). Norepinephrine: not too much, too long. Shock.

[11] Sacha GL, Lam SW, Duggal A, Torbic H, Bass SN, Welch SC, Butler RS, Bauer SR (2018). Predictors of response to fixed-dose vasopressin in adult patients with septic shock. Ann Intensive Care.

[12] Bassi E, Park M, Azevedo LC (2013). Therapeutic strategies for high-dose vasopressor-dependent shock. Crit Care Res Pract.

[13] Correa TD, Takala J, Jakob SM (2015). Angiotensin II in septic shock. Crit Care.

[14] Chawla LS, Busse L, Brasha-Mitchell E, Davison D, Honiq J, Alotaibi Z, Seneff MG (2014). Intravenous angiotensin II for the treatment of high-output shock (ATHOS trial): a pilot study. Crit Care.

[15] Khanna A, English SW, Wang XS, Ham K, Tumlin J, Szerlip H, Busse LW, Altaweel L, Albertson TE, Mackey C, McCurdy MT, Boldt DW, Chock S, Young PJ, Krell K, Wunderink RG, Ostermann M, Murugan R, Gong MN, Panwar R, Hastbacka J, Favory R, Venkatesh B, Thompson BT, Bellomo R, Jensen J, Kroll S, Chawla LS, Tidmarsh GF, Deane AM (2017). ATHOS-3 investigators: angiotensin II for the treatment of vasodilatory shock. N Engl J Med.

[16] Fliser D, Arnold U, Kohl B, Hartung R, Ritz E (1993). Angiotensin II enhances insulin sensitivity in healthy volunteers under euglycemic conditions. J Hypertens.

[17] Wolf RL, Mendlowitz M, Gitlow SE, Naftchi N (1961). Metabolism of angiotensin II-I-131 in normotensive and hypertensive human subjects. Circulation.

[18] Fliser D, Dikow R, Demukaj S, Ritz E (2000). Opposing effects of angiotensin II on muscle and renal blood flow under euglycemic conditions. J Am Soc Nephrol.

[19] Dusterdieck G, McElwee G (1971). Estimation of angiotensin II concentration in human plasma by radioimmunoassay. Some applications to physiological and clinical states. Eur J Clin Invest.

[20] Bucher M, Hobbhahn J, Kurtz A (2001). Nitric oxide-dependent down-regulation of angiotensin II type 2 receptors during experimental sepsis. Crit Care Med.

[21] Bucher M, Ittner KP, Hobbhahn J, Taeger K, Kurtz A (2001). Downregulation of angiotensin II type 1 receptors during sepsis. Hypertension.

[22] Chawla LS, Chen S, Bellomo R, Tidmarsh GF (2018). Angiotensin converting enzyme defects in shock: implications for future therapy. Crit Care.

