# Supplementary material for: Sensitivity to angiotensin II dose in patients with vasodilatory shock: a prespecified analysis of the ATHOS-3 trial
Source: Ann Intensive Care. 2019 Jun 3;9:63. doi: 10.1186/s13613-019-0536-5 (PMC6546778; doi:10.1186/s13613-019-0536-5)
Supplement: Supplementary file 1 — Additional file 1: Table S1. Summary of Treatment-Emergent Adverse Events (regardless of causality) Reported in >5% of Patients in Either Dosing Subgroup. [file 13613_2019_536_MOESM1_ESM.docx]

**SUPPLEMENTAL MATERIAL**

| **Table S1.** Summary of Treatment-Emergent Adverse Events (regardless of causality) Reported in >5% of Patients in Either Dosing Subgroup | | |
| --- | --- | --- |
|  | **Angiotensin II >5 ng•kg^−1^•min^−1^ (n = 84)** | **Angiotensin II ≤5 ng•kg^−1^•min^−1^ (n = 79)** |
| Multiorgan failure | 13 (15.5) | 12 (15.2) |
| Hypokalemia | 3 (3.6) | 10 (12.7) |
| Hypotension | 7 (8.3) | 10 (12.7) |
| Thrombocytopenia | 6 (7.1) | 10 (12.7) |
| Atrial fibrillation | 13 (15.5) | 9 (11.4) |
| Delirium | 2 (2.4) | 7 (8.9) |
| Acute kidney injury | 2 (2.4) | 6 (7.6) |
| Anemia | 6 (7.1) | 6 (7.6) |
| Bradycardia | 2 (2.4) | 5 (6.3) |
| Constipation | 0 | 5 (6.3) |
| Agitation | 2 (2.4) | 4 (5.1) |
| Anxiety | 0 | 4 (5.1) |
| Decubitus ulcer | 2 (2.4) | 4 (5.1) |
| Hypophosphatemia | 2 (2.4) | 4 (5.1) |
| Hypoxia | 0 | 4 (5.1) |
| Respiratory failure | 5 (6.0) | 4 (5.1) |
| Pleural effusion | 6 (7.1) | 3 (3.8) |
| Septic shock | 15 (17.9) | 3 (3.8) |
| Tachycardia | 6 (7.1) | 2 (2.5) |
